# 3,4-Dihydroxybenzalacetone (DBL) Prevents Aging-Induced Myocardial Changes in Senescence-Accelerated Mouse-Prone 8 (SAMP8) Mice

**DOI:** 10.3390/cells9030597

**Published:** 2020-03-03

**Authors:** Vijayasree V. Giridharan, Vengadeshprabhu Karupppagounder, Somasundaram Arumugam, Yutaka Nakamura, Ashrith Guha, Tatiana Barichello, Joao Quevedo, Kenichi Watanabe, Tetsuya Konishi, Rajarajan A. Thandavarayan

**Affiliations:** 1 Translational Psychiatry Program, Faillace Department of Psychiatry and Behavioral Sciences, McGovern Medical School, The University of Texas Health Science Center at Houston (UTHealth), Houston, TX 77054, USA; vijayasree.v.giridharan@uth.tmc.edu (V.V.G.); Tatiana.barichello@uth.tmc.edu (T.B.); Joao.L.DeQuevedo@uth.tmc.edu (J.Q.); 2 Faculty of Applied Life Sciences, Faculty of Pharmaceutical Sciences, Niigata University of Pharmacy and Applied Life Sciences Niigata, Niigata 956-8603, Japan; nakamura@nupals.ac.jp; 3 Department of Orthopedics and Rehabilitation, Penn State College of Medicine, 500 University Drive, Hershey, PA 17033, USA; vkaruppagounder@pennstatehealth.psu.edu; 4 Department of Clinical Pharmacology, Faculty of Pharmaceutical Sciences, Niigata University of Pharmacy and Applied Life Sciences, 265-1, Higashijima, Akiha ku, Niigata 956-8603, Japan; somasundaram143@rediffmail.com (S.A.); wataken@med.niigata-u.ac.jp (K.W.); 5 National Institute of Pharmaceutical Education and Research (NIPER), Jadavpur, Kolkata, West Bengal 700032, India; 6 Department of Cardiology, Houston Methodist Hospital, Houston, TX 77030, USA; gashrith@houstonmethodist.org; 7 Center of Excellence on Mood Disorders, Faillace Department of Psychiatry and Behavioral Sciences McGovern Medical School, The University of Texas Health Science Center at Houston (UTHealth), Houston, TX 77054, USA; 8 Neuroscience Graduate Program, The University of Texas MD Anderson Cancer Center UTHealth, Graduate School of Biomedical Sciences, Houston, TX 77030, USA; 9 Translational Psychiatry Laboratory, Graduate Program in Health Sciences, University of Southern Santa Catarina (UNESC), Criciúma 88800-000, SC, Brazil; 10 Department of Laboratory Medicine and Clinical Epidemiology for Prevention of Noncommunicable Diseases, Niigata University Graduate School of Medical and Dental Sciences, 757, Ichibancho, Asahimachidori, Chuo ku, Niigata City 951-8510, Japan; 11 Niigata University of Pharmacy & Applied Life Sciences (NUPALS), LIAISON R/D Center, Niigata 956-8603, Japan

**Keywords:** 3,4-dihydroxybenzalacetone, SAMP8, DNA damage, fibrosis, apoptosis

## Abstract

Aging is a predominant risk factor for the development and progression of cardiovascular complications. Physiologically and anatomically, the heart undergoes numerous changes that result in poor cardiac function in the elderly population. Recently, several studies have provided promising results, confirming the ability of the senescence-accelerated mouse-prone 8 (SAMP8) model to accurately model age-related cardiovascular alterations. In this study, using a murine model of senescence, SAMP8, we aimed to investigate the effect of 3,4-dihydroxybenzalacetone (DBL), a catechol-containing phenylpropanoid derivative isolated from *Inonotus obliquus* (Chaga), on cardiac aging. DBL was administered at the doses of 10 mg/kg and 20 mg/kg by oral gavage to SAMP8 mice to examine aging-mediated cardiac changes, such as oxidative DNA damage, oxygen radical antioxidant capacity (ORAC) value, fibrosis, inflammation, and apoptosis. The treatment with DBL at both doses significantly reduced aging-mediated oxidative DNA damage, and simultaneously increased the ORAC value in the SAMP8 assay. Cardiac fibrosis was assessed with Azan-Mallory staining, and the number of cardiac remodeling markers was found to be significantly reduced after the treatment with DBL. We also observed a decrease in cardiomyocyte apoptosis as measured by the terminal transferase-mediated dUTP nick end labeling (TUNEL) staining method and the caspase-3 levels in SAMP8 mice compared with senescence-resistant control (SAMR1) mice. The findings from this study suggest that DBL has a potentially beneficial effect on aging-mediated myocardial alterations. Further studies are warranted to confirm the promising potential of this catechol compound against aging-associated myocardial dysfunction.

## 1. Introduction

Aging is an inevitable, complex biological phenomenon that all living organisms experience. Advancements in health sciences have steadily led to increased longevity, which has significantly increased the elderly population [1]. Similar to other organs, the myocardium undergoes structural and functional modifications during aging. Cardiac aging is characterized by cellular and molecular alterations in the heart that may include increased mitochondrial oxidative stress, fibrosis, inflammation, and apoptosis [2]. Aging-induced cardiac fibrosis may affect both myocardial relaxation and contractility. The loss of cardiomyocytes through apoptosis is a crucial event underlying the development of cardiac dysfunction, which is evident in the progression of cardiac dysfunction. In addition, there is a senescence-induced inflammation that is sustained through the upregulation of the release of cytokines, leading to the proliferation of fibroblasts an activation of metalloproteinases [3]. Deciphering the molecular mechanisms underlying myocardial dysregulation is important for identifying the targets to potentially intervene and attenuate the degenerative processes in cardiac senescence.

The compound 3,4-dihydroxy benzal acetone (DBL) is a catechol that contains a phenylpropanoid derivative, an isolated compound from *Inonotus obliquus* (Chaga). *Inonotus obliquus* is a fungus that is found in Russia, Scandinavia, Central Europe, and Eastern Europe [4]. In Russia, it is called Chaga and it has been used as a traditional medicine with many potential health benefits, particularly the modulation of the immune system. From its fruiting body, approximately seven small phenolic compounds were isolated—4-hydroxy-3,5-dimethoxy benzoic acid 2-hydroxy-1-hydroxymethyl ethyl ester (BAEE), protocatechic acid (PCA), caffeic acid (CA), 3,4-dihybenzaladehyde (DB), 2,5-dihydroxyterephtalic acid (DTA), syringic acid (SA), and DBL [5]. The low-molecular-weight polyphenol DBL has been reported to inhibit the activity of human topoisomerase (topo) I and II. Previous research revealed its ability to suppress the proliferation of human cancer cells by arresting the cell cycle through the inhibition of the cellular activity of topo II [6]. In vitro studies have demonstrated the neuroprotective potential of DBL against Parkinson’s disease-related neurotoxin 6-hydroxydopamine in SH-SY5Y cells through the activation of the Nrf2/glutathione pathway. Notably, Nrf2 dysfunction exacerbates the pathogenesis of age-related vascular disease and promotes cellular senescence [7,8]. In another in vitro study, a pre-treatment with DBL was shown to inhibit the lipopolysaccharide (LPS) and induced NF-κB signaling, activating its downstream signaling genes NOS2, interleukin (IL)1-β, and IL-6 [9]. DBL was also shown to inhibit H_2_O_2_-induced oxidative stress by selectively inhibiting p38-MAPK signaling [10]. 

Several studies have proposed senescence-accelerated mouse-prone 8 (SAMP8) as a suitable model to evaluate cardiac aging. A study by Forman et al. demonstrated that the messenger RNA (mRNA) and protein levels of inflammatory cytokines, such as IL-1 and tumor necrosis factor (TNF)-α, were elevated in SAMP8 mice heart, with a simultaneous reduction in IL-10 expression. The levels of the oxidative stress marker heme oxygenase (HO)-1 and apoptosis markers BAX (BCL2-Associated X Protein), caspase-3, and caspase-9 were also elevated in SAMP8 mice heart [11]. We recently demonstrated that aging-mediated myocardial changes increased endoplasmic reticulum stress and the role of macrophage polarization in cardiac remodeling, using the SAMP8 mice model [12,13]. Thus, the aging-induced alteration in oxidative stress, inflammation, cardiac remodeling, and apoptosis is significantly recapitulated in this mice model [14]. Hence, in the present study, we used SAMP8 as a model for cardiac aging, and we investigated the cardioprotective effect of DBL. Aging-mediated myocardial changes, such as oxidative DNA damage, fibrosis, inflammation, and apoptosis, were measured after treatment with DBL, and the results were compared with those obtained with senescence-resistant control (SAMR1) mice.

## 2. Materials and Methods

### 2.1. Animals

SAMP8 and SAMR1 male mice were provided by Japan SLC Inc., and were maintained under standard conditions (temperature 23 ± 1 °C, humidity 50–60%, 12:12-h light-dark cycle, lights on at 7:00 a.m.), with food in the form of dry pellets, and tap water available ad libitum throughout the study. The animal experiments were performed according to internationally followed ethical standards, and were approved by the Niigata University of Pharmacy and Applied Life Sciences. The approval number for this protocol was: H220401.

### 2.2. Acute Oral Toxicity

Male C57BL/6 JAX mice were obtained from Charles River Japan Inc., Kanagawa, Japan. Mice, aged 6 to 8 weeks, weighing 15 to 20 g, were used. We performed the acute oral toxicity studies according to the procedure and guidelines described by the Organization for Economic Co-operation and Development (OECD). We followed the OECD 407 guidelines to conduct acute oral toxicity studies [15]. DBL was dissolved in 1% of ethanol in saline, at the doses of 10, 100, and 1000 mg/kg of body weight, and it was given in single oral doses to mice that had been starved overnight. The mice were observed individually after dosing, at least once during the first 30 min, periodically during the first 24 h, and daily thereafter, for a total of 14 days. There were no adverse clinical signs and no mortality among the animals subjected to the acute oral doses. We then selected the doses of 10 and 20 mg/kg for the treatment.

### 2.3. Drugs and Chemicals

The synthesis of DBL was carried out at the Niigata University of Pharmacy and Applied Life Sciences, as described previously [7]. The yellow DBL was evaluated by HPLC and the purity was 99.9%. Acetylthiocholine iodide (AChI) and 5,5’-dithiobis (2-nitrobenzoic acid) (DTNB) were purchased from Sigma-Aldrich (St. Louis, MO, USA). All other chemicals used in the study were of analytical grade. Solutions of the drug and chemicals were freshly prepared before use.

### 2.4. Experimental Design

The experimental groups were SAMR1, SAMP8, SAMP8 + DBL 10 mg/kg, and SAMP8 + 20 mg/kg. DBL was administered orally for 90 days at the doses of 10 mg/kg and 20 mg/kg to SAMP8 mice. The SAMR1 mice were given 0.1% ethanol in saline for 90 days. On day 90, the mice were anesthetized (using pentobarbital, 50 mg/kg, intraperitoneally (i.p.)). Using tail vein puncture, blood was collected and centrifuged at 2000× *g* for 5 min at 4 °C, and the resultant plasma was frozen at –80 °C. The mice were then decapitated, and the heart tissue was quickly removed, blotted gently with filter paper to remove the blood and extraneous tissues, then frozen in liquid nitrogen and stored at −80 °C until use.

### 2.5. Oxygen Radical Absorbing Capacity (ORAC)

The ORAC was measured using disodium fluorescein as a fluorescence probe, and 2,2-azobis (2-amidinopropane) dihydrochloride (AAPH) as a peroxyl radical generator. The plasma samples were run in the same plate as the standard (Trolox) and control samples (Fluorescein alone and fluorescein plus). The measurement of ORAC was considered relevant due to the abundance of the peroxyl free radicals in biological systems [16,17].

### 2.6. Alkaline Single-Cell Gel Electrophoresis Method or Comet Assay—DNA Damage Assay

The standard protocol for the comet assay preparation and analysis implemented was based on previously published results [18,19,20]. The heart was quickly excised after deep anesthesia. The isolated cardiomyocytes, as described previously [21], were added to a 0.8% low-melting agarose solution, prepared in 0.9% NaCl at 38 °C. The suspension was then poured onto fully frosted microscope slides, and the cells were lysed with incubation in a lysis buffer (2.5M NaCl, 100 mM Na 2-EDTA, 10% DMSO, and 0.1% Triton X-100) for 1 h at 4 °C. For equilibration, the slides were transferred to an electrophoretic tank containing an alkaline solution (300 mM NaOH, 1 mM EDTA, pH 13.0) for 20 min. Electrophoresis was performed for 30 min at 25 V, and the slides were washed gently in a neutralizing buffer (0.4 M Tris-HCl, pH 7.5) and stored at 4 °C until the observation. The slides were stained with SYBR Green II, and at least 50 cells were captured per slide at 200× magnification using a fluorescence microscope (Olympus (BH2-RFCA), Japan). The captured comet images were analyzed using the digital imaging software CASP [18]. Using the software, parameters such as the tail length, the tail moment, the percentage of DNA within the tail, and the Olive tail moment were quantified to evaluate the extent of DNA damage in SAMP8 and SAMPR1 cardiomyocytes.

### 2.7. Histopathological Examination

The heart tissues were fixed in 10% neutral buffered formalin. The sections were cut to a 3–5 µm thickness and stained with an Azan-Malory (A-M) staining kit for cardiac fibrosis. The analysis was done using a color image analyzer (CIA102; Olympus, Tokyo, Japan) to identify the fibrotic tissue in the control and treated hearts, as previously described [21].

### 2.8. Gene Expression Analysis by Real-Time Reverse Transcription-Polymerase Chain Reaction (RT-PCR)

Heart tissues collected after mice euthanasia were preserved by immersing them in RNAlater (Ambion Inc., Austin, TX, USA). Using a standard protocol, the extraction of total RNA was performed after homogenization using Ultra Turrax T8 (IKA Labortechinik, Staufen, Germany) in a TRIzol reagent (Invitrogen Corp., Carlsbad, CA, USA). The synthesis of cDNA was performed with reverse transcription using total RNA (2 μg) as a template (SuperScript II; Invitrogen Corporation, Carlsbad, CA, USA). The gene expression analysis was performed with RT-PCR (Smart Cycler; Cepheid, Sunnyvale, CA, USA) using cDNA synthesized from the heart specimen. The primers used were transforming growth factor (TGF)-β (Mm01178820_m1), collagen III (Mm01268569_m1), matrix metallopeptidase (MMP)-2 (Mm00439498_m1), MMP-9 (Mm00442991_m1), TNF-α (Mm00443258_m1), and IL-6 (Mm00446190_m1). The real-time RT-PCR was monitored with a TaqMan probe (TaqMan Gene expression assays; Applied Biosystems, Foster City, CA, USA) and performed in accordance with the following protocol: 600 s at 95 °C, followed by thermal cycles of 15 s at 95 °C and 60 s at 60 °C for extension. Relative standard curves produced using several 10-fold dilutions (1:10:100:1000:10,000:100,000) of cDNA from the cortex were used for the linear regression analysis of other samples. We used glyceraldehyde-3-phosphate dehydrogenase (GAPDH) mRNA as an internal control to normalize the results, which are shown as relative mRNA levels.

### 2.9. Immunofluorescence

For the immunofluorescence, heart tissues were fixed in a 10% buffered formaldehyde solution and embedded in paraffin. Paraffin was removed using a xylene wash, then heat-induced antigen retrieval was performed. The sections were then blocked with a 10% goat serum in phosphate-buffered saline for 1 h. The primary antibody anti-caspase-3 (1:200) (Santa Cruz Biotechnology, Santa Cruz, CA, USA) was incubated overnight at 4 °C. The sections were then incubated with a fluorescein-isothiocyanate-conjugated secondary antibody (1:500) (Sigma-Aldrich, St. Louis, MO, USA). Samples were visualized with a fluorescence microscope at 400× magnification (CIA-102; Olympus, Tokyo, Japan).

### 2.10. Analysis of Myocardial Apoptosis by Terminal-Transferase-Mediated Dutp Nick-End Labeling (TUNEL) Assays

Frozen heart tissues were embedded in an optimal cutting temperature (OCT) compound and the samples were then cryostat-sectioned at a 4-μm thickness. The sections were then fixed in 4% paraformaldehyde (pH 7.4) at room temperature. The TUNEL assay was performed as specified in the in situ apoptosis detection kit (Takara Bio Inc, Shiga, Japan). Digital photomicrographs were obtained using fluorescence microscopy at 200× magnification (CIA-102, Olympus, Tokyo, Japan). Five sections were scored per sample to evaluate the apoptotic nuclei. Ten fields were randomly chosen from each slide and, using a defined rectangular field area, a total of 100 cells per field were counted. The percentage of total myocytes that were TUNEL-positive (apoptotic index) was then calculated. This evaluation was performed in a blinded manner.

### 2.11. Statistical Analysis

The statistical difference between the groups was determined with a one-way analysis of variance (ANOVA) followed by Tukey’s method. The differences were considered statistically significant at *p* < 0.05. The data are presented as mean ± standard error (S.E.).

## 3. Results

### 3.1. Antioxidant Potential of the Treatment with DBL as Measured by the ORAC Assay

**Figure 1:**
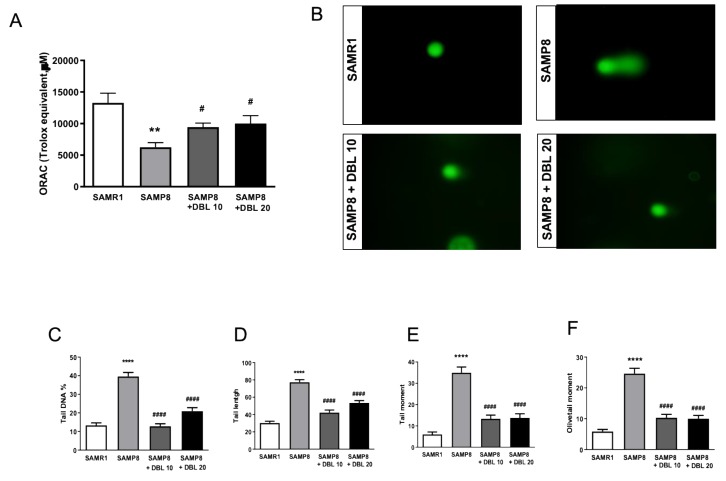
(**A**) The antioxidant capacity of 3,4-dihydroxybenzalacetone (DBL) as measured by an oxygen radical antioxidant capacity (ORAC) assay. The reduction of aging-induced oxidative DNA damage after DBL treatment (**B**–**F**): **B**. The representative photomicrographs of typical comet figures stained with SYBR Green-II. The DNA damage was detected by a single-cell gel electrophoresis assay (comet assay) in cardiomyocytes of all groups at 200× magnification. The quantification of cardiomyocyte DNA damage analyzed by digital imaging casp-software. The tail DNA percentage, **D**. The tail length, **E**. The tail moment, and **F**. the Olive tail moment. The data represent the mean ± standard error of the mean (SEM). ** *p* < 0.01, **** *p* < 0.0001 vs. the group of senescence-resistant control (SAMR1) mice; ^#^ *p* < 0.05, ^####^ *p* < 0.0001 vs. senescence-accelerated mouse-prone 8 (SAMP8) mice, using a one-way analysis of variance (ANOVA) with Tukey’s post-test.

Using the ORAC assay, we measured the capacity of the treatment with DBL to retard the free-radical-induced loss of fluorescence. The ORAC assay is widely used to measure the antioxidant potential of food and natural products. As shown in Figure 1A, the antioxidant capacity of DBL was assessed by the ORAC value, where one ORAC unit equals the fluorescence decay inhibited by 1 μM of Trolox (standard). These data demonstrate that the treatment with DBL increased the ORAC value in the plasma of SAMP8 mice, and thus protected against aging-induced free radical generation.

### 3.2. Treatment with DBL Protects the Cardiomyocytes from Aging-Induced DNA Damage as Measured by Comet Assay

One possible explanation for aging cardiomyocytes includes the age-related oxidative DNA damage. To assess the oxidative DNA damage in single cells, the alkaline comet assay serves as a method of evaluation [22,23]. As demonstrated in Figure 1B–F, we found a significant increase in tail DNA percentage, tail length, tail moment, and Olive moment in SAMP8 cardiomyocytes compared with control SAMR1 mice, confirming the extent of the DNA damage. The treatment with the polyphenol DBL (10 mg/kg and 20 mg/kg) significantly reduced the tail DNA percentage, tail length, tail moment, and Olive moment in SAMP8 mice.

### 3.3. DBL Prevents Aging-Induced Fibrotic Changes in the SAMP8 Mice Hearts

**Figure 2:**
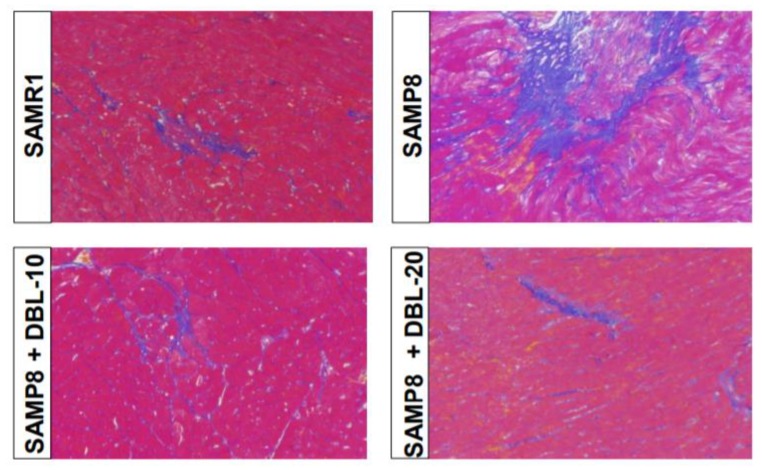
The regression of the aging-induced fibrosis after treatment with DBL. The images of Azan-Mallory (A-M) stained heart sections of SAMR1 and SAMP8 mice depict cardiac fibrosis (blue area).

A classic histological feature of cardiac aging is myocardial fibrosis. Evidence from both preclinical and clinical studies has demonstrated that the aging heart undergoes fibrotic remodeling [24]. As shown in Figure 2, we observed increases in cardiac fibrosis in SAMP8 mice hearts, as illustrated by the blue scar, which indicates an elevated level of fibrotic formation. A decreased level of fibrotic formation was observed in the SAMP8 heart when treated with DBL.

### 3.4. DBL Prevents Myocardial Apoptosis in the SAMP8 Mice Hearts

**Figure 3:**
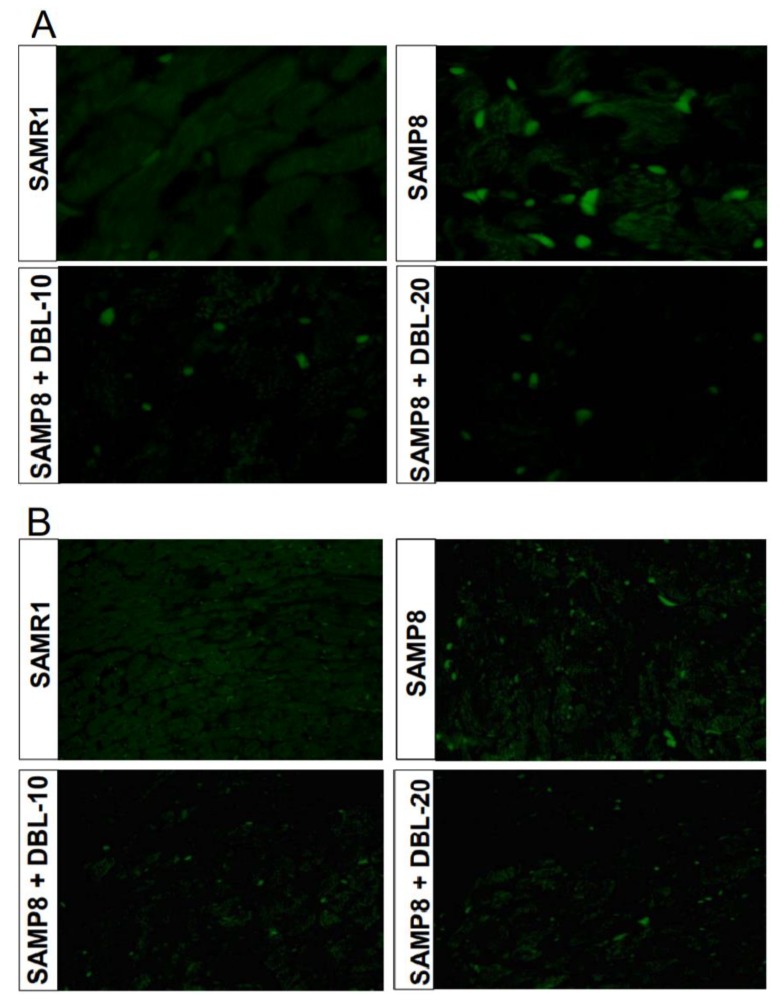
The treatment with DBL inhibits aging-mediated caspase-3 positive cells and cardiac apoptosis. (**A**). The representative photomicrographs of heart sections show the caspase-3 immunofluorescence identified by immunohistochemical staining with anti-caspase-3 antibodies at 400× magnification. (**B**). The representative photomicrographs show myocardial tissue sections terminal transferase-mediated dUTP nick end labeling (TUNEL)-stained for the detection of apoptotic nuclei at 200× magnification.

Myocardial apoptosis plays a crucial role in cardiac output and function as aging initiates the loss of cardiomyocytes through programmed cell death [25,26]. We measured the level of apoptosis by evaluating the caspase-3 expression and TUNEL-positive nuclei using immunofluorescence. As shown in Figure 3, the results revealed that an aging-induced increase in apoptosis was evident in SAMP8 mice, as illustrated by the elevated caspase-3 expression and TUNEL-positive nuclei. After the treatment with DBL, the expression of caspase-3 and TUNEL-positive nuclei was reduced in the SAMP8 model.

### 3.5. DBL Modulates Myocardial Inflammatory Cytokines and Cardiac Remodeling Gene Expression

**Figure 4:**
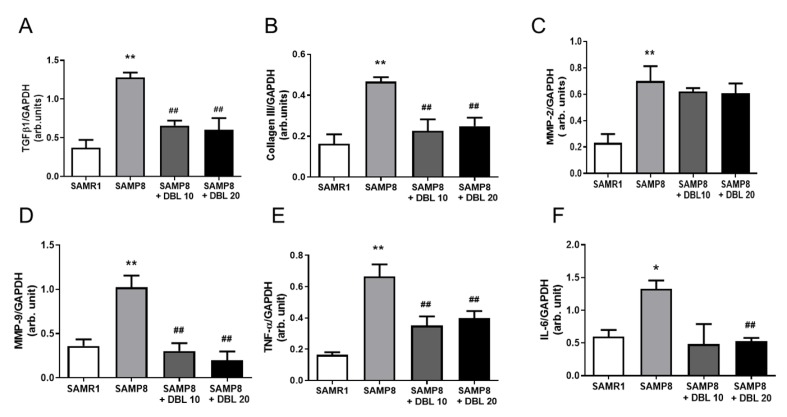
DBL modulates the aging-induced messenger RNA expression of inflammatory and cardiac remodeling marker genes in the heart tissue. (**A**–**F**). The graphs show the messenger RNA (mRNA) expression levels of **A**. Transforming growth factor (TGF)-β1, **B**. Tumor necrosis factor (TNF)-α, **C**. Interleukin (IL)-6, **D**. Matrix metallopeptidase (MMP)-2, **E**. MMP-9, and **F**. Collagen 1. The data represent the mean ± SEM. ** *p* < 0.01 vs. SAMR1, ^##^ *p* < 0.01 vs. SAMP8, using a one-way ANOVA followed by Tukey’s post hoc test.

To further understand the aging-induced changes, the gene expression of various inflammatory markers and cardiac remodeling markers was examined with RT-PCR analysis. As demonstrated in Figure 4, the SAMP8 group had significantly increased levels of TNF-α, TGF-β1, MMP-2, MMP-9, and collagen-III, compared with the SAMR1 group. The treatment with DBL at both doses significantly downregulated the gene expression of TNF-α, TGF-β1, and MMP-9, and reduced the levels of collagen-III.

## 4. Discussion

The average human life expectancy doubled over the last 200 years in developing countries. Even though life expectancy has improved, an increase in longevity enhances the risk of chronic illnesses such as cancer, cardiovascular diseases, and neurodegenerative diseases in the elderly [1]. According to the WHO, approximately 17.9 million people die from cardiovascular diseases each year, which accounts for 31% of deaths worldwide [27]. According to reports from the American Heart Association (AHA), the incidence of cardiovascular diseases increases with age. The risk is approximately 75% in individuals aged 60–79 years, and 86% above the age of 80 years. Thus, aging-induced cardiovascular disease is one of the major burdens for the United States’ healthcare system [28]. In addition to understanding the etiologies associated with cardiac aging, alternative approaches are required in order to meet the needs of the aging population. Hence, in this study, we investigated the effect of DBL, an isolated compound from *Inonotus obliquus,* a medicinal mushroom, on aging-mediated oxidative stress, inflammation, and apoptosis, using the SAMP8 heart as a model of cardiac aging.

We used the accelerated aging model senescence-accelerated mouse (SAM), which was developed through the phenotypic selection of the AKR/J strain of mice [29]. SAMP8 mice also demonstrate the presence of age-related alterations in the heart, liver, lung, and skin [30,31,32,33]. Recently, we have demonstrated the potential factors involved in a cardiac malfunction in SAMP8, and they include increased endoplasmic reticulum stress, alteration in the HMGB1-TLR2/TLR4 signaling cascade, as well as the induction of a phenotypic switch from the polarization of M1 macrophages in SAMP8 heart [12,13]. This study evaluates DBL as a treatment for cardiac aging utilizing the SAMP8 model.

One of the main features of aging is the disturbance in the balance between the free radical generation and the antioxidant defense in the body. Globally, emerging research suggests that antioxidants increase the state of healthy aging and longevity by interfering with the generation of reactive oxygen species (ROS), or by inhibiting the formation of ROS [34,35]. At the cellular level, an increase in ROS leads to cellular senescence, which is further responsible for the ensuing apoptosis, necrosis, and autophagy in organismal aging [36]. Hence, we tested the antioxidant capacity of DBL by performing an ORAC assay. The plasma from DBL-treated groups demonstrated elevated levels of ORAC value measured in terms of Trolox equivalent. An increased ORAC value represents the antioxidant capabilities of DBL. A classic hallmark of aging includes oxidative DNA damage. To evaluate the potential of DBL for preventing age-dependent DNA damage, we performed a single-cell alkaline phosphatase assay. The isolated cardiomyocytes from the SAMP8 hearts had increased tail length and tail moment. The treatment with DBL at both doses significantly reduced the comet length. Similar to these results, the treatment with DBL increased the expression of antioxidant genes, such as HMOX1, GCLM, and NQO1, in human SH-SY5Y cells [9]. Similarly, the antioxidant potential of other chemical compounds isolated from Chaga has been compared with the standard antioxidant butylated hydroxyanisole (BHA) and Trolox [37]. Cardiac fibroblasts play a key role in establishing the homeostasis of the myocardial extracellular matrix. During cardiac aging and insult, fibroblasts transform into myofibroblasts, leading to myocardial fibrosis [38]. The aging-induced increase in myofibroblasts was observed as cardiac fibrosis, as illustrated by a blue area in the A-Z staining in SAMP8 mice. We also observed an increase in the major component of the myocardial extracellular matrix, the collagen. Collagen III is the most abundant subtype in the cardiac tissue. Thus, we examined the expression of mRNA, and it was increased in the SAMP8 group of mice. Increased levels of collagen III and cardiac fibrosis in the 6-month-old SAMP8 mouse model have been previously reported by Reed et al. [39] and this is consistent with our findings [40]. Interestingly, the treatment with DBL significantly downregulated the levels of collagen III mRNA in the SAMP8 mouse group, and decreased the aging-mediated fibrosis formation in the SAMP8 hearts. We also measured mRNA levels of MMP-2 and MMP-9, as these have been reported to be the major metalloproteinases responsible for the extracellular matrix degradation in the heart, and their expression has been reported to increase with age [41]. As expected, the SAMP8 aging model had elevated MMP-9 levels and chronic treatment with DBL significantly reduced the levels of MMP-9 in SAMP8 hearts, while no significant change was observed in the expression of MMP-2. Given the role of p53 activation in cellular senescence, we found that DBL effectively inhibited the expression of p53 in the brain in an aging model (unpublished data).

We also investigated the effect of DBL on the expression of proinflammatory cytokines, as many cardioprotective compounds inhibit inflammatory cytokines [42,43]. Elevated levels of the proinflammatory cytokines TGF-β, TNF-α, and IL-6 mRNA were also observed in SAMP8 mice. However, the treatment with DBL at both doses significantly decreased the elevated levels of mRNA in SAMP8 mice. Cardiomyocyte apoptosis has also been demonstrated in clinical and preclinical studies to contribute to congestive heart failure [44]. In this study, we observed that treatment with DBL effectively diminished the aging-mediated apoptosis, as demonstrated by a reduction in the TUNEL-positive nuclei and in caspase-3 levels.

In conclusion, the present study demonstrated the in vivo cardioprotective potential of DBL against age-associated cardiac changes, through the inhibition of oxidative stress, inflammation, and apoptosis in a SAM model. However, further research is warranted to understand the molecular mechanism by which DBL provides cardiac protection. We plan to further explore the inhibitory potential of DBL in the context of cellular senescence in cardiac aging.
